# Seaweed-Derived Alginate–Cellulose Nanofiber Aerogel for Insulation Applications

**DOI:** 10.1021/acsami.1c07954

**Published:** 2021-07-13

**Authors:** Linn Berglund, Tuukka Nissilä, Deeptanshu Sivaraman, Sanna Komulainen, Ville-Veikko Telkki, Kristiina Oksman

**Affiliations:** † Division of Materials Science, Luleå University of Technology, SE 971 87 Luleå, Sweden; ‡ Fiber and Particle Engineering Research Unit, University of Oulu, FI 90570 Oulu, Finland; § Empa—Building Energy Materials and Components, Swiss Federal Laboratories for Materials Science and Technology, CH 8600 Dübendorf, Switzerland; ∥ NMR Research Unit, University of Oulu, FI 90570 Oulu, Finland; ⊥ Mechanical & Industrial Engineering, University of Toronto, Toronto, Ontario M5S 3G8, Canada

**Keywords:** cellulose, nanofibers, brown seaweed, alginate, aerogels, insulation materials

## Abstract

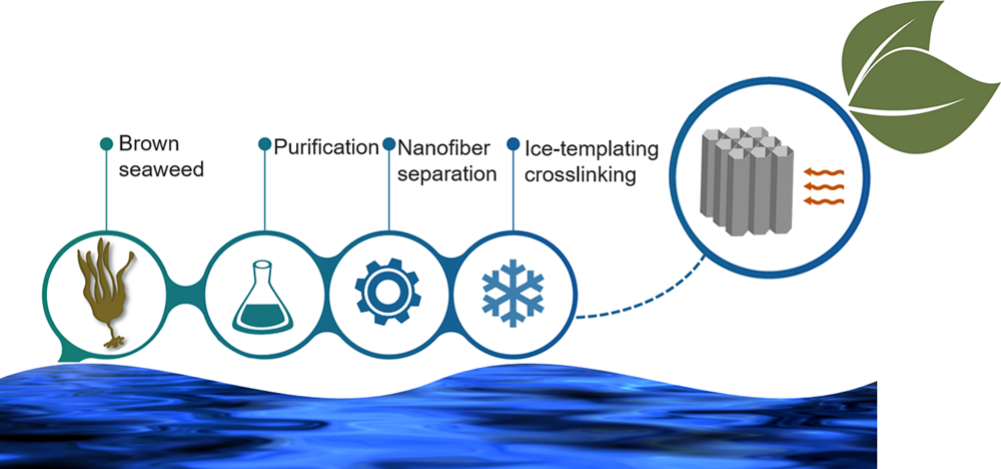

The next generation of green insulation materials is being developed to provide safer and more sustainable alternatives to conventional materials. Bio-based cellulose nanofiber (CNF) aerogels offer excellent thermal insulation properties; however, their high flammability restricts their application. In this study, the design concept for the development of a multifunctional and non-toxic insulation material is inspired by the natural composition of seaweed, comprising both alginate and cellulose. The approach includes three steps: first, CNFs were separated from alginate-rich seaweed to obtain a resource-efficient, fully bio-based, and inherently flame-retardant material; second, ice-templating, followed by freeze-drying, was employed to form an anisotropic aerogel for effective insulation; and finally, a simple crosslinking approach was applied to improve the flame-retardant behavior and stability. At a density of 0.015 g cm^–3^, the lightweight anisotropic aerogels displayed favorable mechanical properties, including a compressive modulus of 370 kPa, high thermal stability, low thermal conductivity (31.5 mW m^–1^ K^–1^), considerable flame retardancy (0.053 mm s^–1^), and self-extinguishing behavior, where the inherent characteristics were considerably improved by crosslinking. Different concentrations of the crosslinker altered the mechanical properties, while the anisotropic structure influenced the mechanical properties, combustion velocity, and to some extent thermal conductivity. Seaweed-derived aerogels possess intrinsic characteristics that could serve as a template for the future development of sustainable high-performance insulation materials.

## Introduction

The design of green insulation materials includes the use of renewable, bio-based raw materials and their eco-efficient and sustainable processing into next-generation building materials. Today, majority of commercially available flame retardants used in insulation are derived from unsustainable petroleum resources, chemically modified organophosphorus, organonitrogen, and halogenated organic compounds.^1^ Although they are highly effective in suppressing flammability, their harmful environmental impact and risk to human health have led to restrictions on their use.^2^ Additionally, some bio-based insulation materials such as hemp and cellulose fibers contain relatively large amounts of additives such as flame-retardant salts, impregnating substances, or binders that are not bio-based. Furthermore, their insulating performance cannot compete with the commercially available insulation materials, such as expanded polystyrene (EPS) and polyurethane (PU) foams.^3^

Aerogels are promising candidates in the field of thermal super-insulation^4,5^ and can be inorganic (silica-based)^6^ or organic (e.g., resorcinol–formaldehyde).^7^ However, they are usually relatively expensive, fragile (silica aerogel),^7^ or toxic (organic aerogels).^8^ Aerogels derived from biodegradable and renewable resources are thus highly desired, and when synthesized in combination with green chemistry principles, they are promising as next-generation, environmentally friendly insulation and flame-retardant materials.

Extracted from abundant biomass or waste sources, cellulose nanofiber (CNF)-based aerogels offer excellent thermal insulation properties^9^ and are non-toxic, renewable, and biodegradable.^10^ The insulation performance of CNF aerogels strongly depends on the pore structure, alignment, and axis in which insulation is measured, which determines the heat-transfer process in the materials.^11,12^ For example, Sakai et al.^13^ investigated an approach to suppress the thermal diffusion of air in highly porous aerogels by using different drying techniques to control the pore size. Creating microscale spaces with distinct sections was more effective than direct blockage of the air path at the nanoscale, where the pore structure further dictates the mode of heat transfer.^13^ By utilizing the anisotropic properties of the fibrils in an aerogel structure, the thermal conductivity has been shown to be reduced, improving the insulation behavior in one direction.^14−16^ However, flammability is one of the main drawbacks of cellulose-based aerogels, which hinders their use in most insulation applications.^17,18^ By their in situ synthesis of magnesium hydroxide nanoparticles, Han et al.^5^ produced a flame-retardant cellulose aerogel that exhibited self-extinguishing behavior within 40 s but at the trade-off of increased thermal conductivity from 56 to 81 mW m^–1^ K^–1^. Farooq et al.^18^ tested different concentrations of sodium bicarbonate as a flame retardant for CNF aerogels, which resulted in a reduced combustion velocity from 5.84 to 0.20 cm s^–1^. The addition of sodium bicarbonate resulted in only a minor increase in thermal conductivity; however, it led to an increase in moisture uptake. Wicklein et al.^14^ reported the impregnation of graphene oxide and clay mineral sepiolite nanorods combined with freeze-drying to obtain strong anisotropic CNF aerogels, which resisted combustion and possessed a low thermal conductivity of 15 mW m^–1^ K^–1^. Köklükaya et al.^19^ also used nanoparticles to suppress the flammability of CNF aerogels by depositing cationic chitosan, anionic poly(vinylphosphonic acid), and anionic montmorillonite clay with a layer-by-layer coating technique. A major drawback of these studies is the complex preparation routes, which limit large-scale production, increase the production cost, and negatively affect the environment.

Alginate, derived from aquatic biomass,^20^ is an inherently flame-retardant material;^17^ is non-toxic, biocompatible, and biodegradable; and can be easily crosslinked.^21^ With a limiting oxygen index (LOI) of 48.0 and a peak heat release rate (PHRR) of 4.99 kW m^–2^, alginate foams have been shown to compare favorably with viscose fiber, which demonstrate an LOI of 20.0 and a PHRR of 168.75 kW m^–2^.^17^ Therefore, alginate is highly promising for the fabrication of low-flammability aerogels, but on the other hand, alginate-based aerogels exhibit poor mechanical properties.^22^

Crosslinking is another design strategy for improving the mechanical properties and thermal stability of aerogels to ensure mechanical robustness and meet fire safety standards. Shang et al.^22^ crosslinked alginate aerogels with CaCl_2_ using a simple post-crosslinking method. The compressive modulus increased almost 4-fold compared to that of the neat alginate. Although the alginate aerogel itself exhibited low flammability, it was further suppressed by combining ionic crosslinking and the addition of inorganic nanoparticles. However, similar to the CNF aerogels, the inclusion of nanoparticles to reduce flammability came at the cost of increasing the thermal conductivity from 25 mW m^–1^ K^–1^ for neat alginate to 42 mW m^–1^ K^–1^.^22^

The development of bio-based aerogel insulation materials that possess multifunctional properties such as mechanical stability, flame retardancy, and low thermal conductivity, combined with using eco-friendly and upscalable production approaches, remains a challenge.

In this study, a multifunctional green insulation material was inspired by the natural composition of seaweed and developed using a three-step approach. First, CNFs separated from alginate-rich seaweed were used to obtain a resource-efficient, fully bio-based, and inherently non-toxic and flame-retardant material. Second, an anisotropic aerogel was assembled using ice-templating combined with freeze-drying with the purpose to achieve a more effective insulation structure in one direction. Finally, a simple crosslinking approach was applied to improve the flame-retardant behavior and stability. The mechanical properties, combustion, and thermal conductivity of the aerogels were tested along (longitudinal) and across (transverse) the freezing direction. The effects of crosslinking on the aerogel structure and properties, such as thermal stability and flammability, were investigated.

## Experimental Section

### Materials

Brown seaweed (*Laminaria digitata*) was provided by Northern Company Co. (Træna, Norway). The seaweed was harvested in May 2017 on the west coast of the North Atlantic Ocean. Fresh seaweed was stored in a freezer before use. Sodium chlorite (NaClO_2_), sodium hydroxide (NaOH), and acetic acid (96% CH_3_COOH) were purchased from VWR (Stockholm, Sweden). For ionic crosslinking, laboratory-grade calcium chloride was used (90 mM CaCl_2_·2H_2_O; Sigma-Aldrich AB, Stockholm, Sweden). All chemicals were used as received, and deionized water was used for all experiments.

### Purification

The stipe part of brown seaweed was purified to remove pigments and impurities while preserving the cellulose and alginate contents. First, the raw material was defrosted at room temperature (RT; 21 ± 1 °C) overnight and subsequently cut into smaller pieces prior to purification by bleaching with NaClO_2_ (1.7%) in an acetate buffer (pH 4.5) at 80 °C for 2 h. After purification, the material was washed until a neutral pH was achieved. The alginate content of the purified material was 46 ± 11 wt %, and the cellulose content was 23 ± 3 wt %.^23^ Optical microscopy was carried out on a Nikon Eclipse LV 100 Pol (Kanagawa, Japan) to study the morphology of the structure after purification.

### Alginate–CNFs

The purified seaweed was further processed in an MKZA6-3 ultrafine friction grinder (Masuko Sangyo Co., Ltd., Kawaguchi, Japan) equipped with coarse silica carbide (SiC) grinding stones to separate the cellulose fraction into nanofibers. The fibrillation process required an energy of 1.0 kW h kg^–1^, and the nanofiber widths were measured as 6 ± 3 nm using atomic force microscopy. The fibrillation process and characterization of the nanofibers were described in detail in our previous study.^23^ After the fibrillation process, the alginate-CNFs (ACNFs) were visualized using a field-emission scanning electron microscope (FEI Magellan 400 XHR-SEM; Hillsboro, OR, USA) at 5 kV. Prior to analysis, the nanofiber samples were coated with platinum using a sputter coater (Leica EM ACE220, Wetzlar, Germany) to avoid charging effects.

### Preparation of Aerogels

ACNF aerogels were prepared by ice templating. A 1.0 wt % water suspension was stirred for 30 min and defoamed with a planetary mixer (THINKY ARE-250, Thinky Corp., Tokyo, Japan) for 3 min prior to being poured inside a polytetrafluoroethylene mold and unidirectionally frozen at a constant cooling rate of 40 °C h^–1^ starting from RT. Cooling was provided by a liquid nitrogen bath, which was in contact with the copper bottom plate of the mold via a copper rod. The temperature of the bottom plate was controlled using a proportional integral derivative-controlled heating element attached to the top part of the copper rod. After complete freezing, the mold was placed inside a freeze-dryer, and a dry aerogel sample was obtained after approximately 4 days. The aerogels were crosslinked according to a post-crosslinking method reported by Shang et al.^22^ The aerogels were immersed in an ethanol/CaCl_2_ solution at RT for 5 h for crosslinking. The solutions were prepared at 1, 5, and 10 wt % CaCl_2_ to obtain different degrees of crosslinking. The samples were then thoroughly rinsed with ethanol to remove CaCl_2_ and dried in a vacuum oven at 24 °C overnight to remove ethanol. The prepared samples are denoted as ACNF and ACNF-(1, 5, 10)X, in which X denotes the crosslinking process at concentrations of 1, 5, and 10 wt %.

### Compression Properties

Uniaxial unconfined compression tests were performed at 25 °C using a dynamic mechanical analyzer (DMA Q800, TA Instruments, New Castle, DE, USA) to study the effect of crosslinking at different concentrations on the mechanical properties. The longitudinal and transverse directions for the ACNF and ACNF-5X aerogels were further studied to evaluate the effect of alignment on the compression behavior of the aerogels. The samples were preloaded with a 0.05 N load and compressed up to a strain of 100% at a strain rate of 10% min^–1^. The compressive modulus (*E*_<5%_) was calculated below a strain of 5% from the initial linear region of the stress–strain curves. The materials were compared based on the stress at 20 and 40% strain (σ_20%_ and σ_40%_, respectively) and by using different crosslinker concentrations at 80% strain (σ_80%_). The aerogel samples had dimensions of 10 × 10 × 10 mm^3^ and were tested 10 times each. The average results were reported.

### Moisture Absorption

The moisture resilience of the aerogels was evaluated before and after crosslinking at different concentrations by exposing samples (2 cm^3^) to 75% relative humidity for 48 h based on the average relative humidity for Europe 2016 reported by ERA-Interim, European Center for Medium-Range Weather Forecasts (ECMWF). The moisture uptake was calculated from the weight gain relative to the weight of the fully dried aerogel samples, and the reported values were based on five separate measurements for each sample.

### Flammability

The flammability of the aerogels was evaluated in a horizontal configuration according to the effect of different crosslinker concentrations. The samples were tested by igniting the aerogels (10 × 10 × 50 mm) using a butane burner in a horizontal configuration, as described in the UL-94 standard. The flame was introduced on the short side of the sample for 5 s, and the test was conducted three times to assess the repeatability. The combustion process was recorded using a digital camera, and the combustion velocity was measured for 60 s. The combustion in the longitudinal and transverse directions for the ACNF and ACNF-5X aerogels was further studied to evaluate the effect of alignment on the flammability of the aerogels.

### Scanning Electron Microscopy

The microstructures of the ACNF and ACNF-5X aerogels were observed using a scanning electron microscope (JEOL JSM 6460LV, Tokyo, Japan) at an acceleration voltage of 15 kV before and after crosslinking. All samples were coated with platinum using a sputter coater (Leica EM ACE220, Wetzlar, Germany) to avoid charging effects.

### X-ray Microtomography

The 3D internal architecture of the ACNF and ACNF-5X aerogels was further reconstructed to evaluate the effect of crosslinking on the aerogel structures using a Zeiss Xradia 510 Versa, Carl Zeiss (Pleasanton, CA, USA), with a 20× objective performing interior tomography with a field of view of 0.56 mm and a voxel size of 0.56 μm. Samples of approximately 4 mm^3^ were scanned with the region of interest positioned precisely in the center of each sample. Scanning was performed with an X-ray tube voltage of 50 kV, an output power of 4 W, and no X-ray filters. A total of 2401 projections with an exposure time of 6 s resulted in a total scan time of 6 h. Reconstruction was performed using filtered back-projection with the Zeiss Scout-and-Scan Reconstructor software (version 11.1). 3D visualization and analysis were performed using Dragonfly Pro Software (ORS).

### Density

The densities of the ACNF and ACNF-5X aerogels were calculated from mass and volume measurements. The dimensions used in the calculation were 5 cm × 5 cm × 2 cm, measured using a digital caliper. The average densities were calculated based on 10 measured samples.

### Surface Area Analysis

The Brunauer–Emmett–Teller (BET) model was used to estimate the surface area of the ACNF and ACNF-5X samples by conducting an N_2_ adsorption test at 77 K using a Gemini VII 2390a surface area analyzer (Micromeritics Instrument Corp., Norcross, GA, USA) after 3 h of degassing at 80 °C.

### Fourier Transform Infrared Spectroscopy

An attenuated total reflectance Vertex 80v Fourier transform infrared (FTIR) spectrometer (Bruker Corp., Billerica, MA, USA) was used to investigate the crosslinking of the aerogel samples. The spectra were recorded in the wavenumber range of 500–4000 cm^–1^.

### Nuclear Magnetic Resonance Spectroscopy

^13^C nuclear magnetic resonance (NMR) measurements were carried out on a 9.4 T Bruker AVANCE III 400 spectrometer (Billerica, MA, USA) equipped with a 5 mm broad-band probe operating at a ^13^C frequency of 100.602 MHz. The measurement of 1.9 wt % alginate gel in water was carried out at 343 K. DMSO-*d*_6_ (at 39.5 ppm) was added as an internal standard. Quantitative ^13^C spectra were recorded using an inverse-gated proton decoupling sequence (Bruker standard sequence “zgig”). The number of scans was 2000, the relaxation delay was 50 s, and the excitation pulse angle was 90°. The line shapes and intensities of the signals were fitted using the Topspin Fit solid NMR program.

### Thermogravimetric Analysis

A TA Instruments TGA-Q500 thermogravimetric analyzer (New Castle, DE, USA) was used to evaluate the thermal stability of the ACNF and ACNF-5X aerogels. The experiments were performed in the ramp mode from RT to 600 °C at 5 °C min^–1^ under a nitrogen atmosphere. The extrapolated onset temperatures of degradation were calculated according to the ISO 11358 standard.

### Thermal Conductivity

The thermal conductivity (λ) was measured using an in-house guarded two-plate device shielded to measure the *Z*-axis uniaxial heat transfer. The produced samples were stored under a controlled atmosphere of 23 °C and 40% humidity for 24 h before measurements (for equilibrium with the atmosphere) were conducted. The temperature difference was maintained at 10 °C using a cold plate at 20 °C and a hot plate maintained at 30 °C. Each sample was measured for 1 h with calibrations for equilibration and steady-state heat flow in the software.^24^

## Results and Discussion

**Figure 1:**
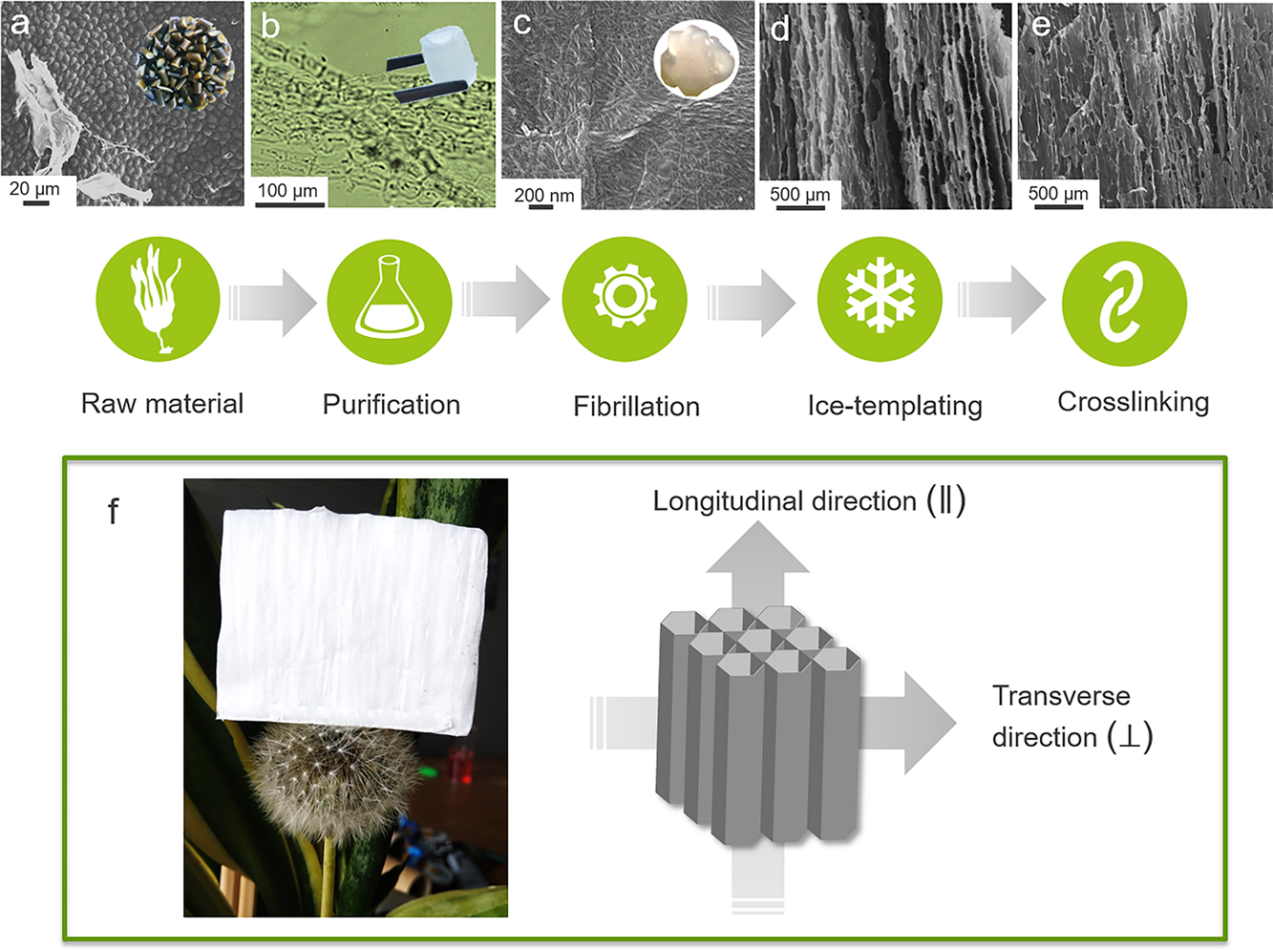
Schematic and microscopic images at the different processing steps: (a) raw material (brown seaweed stipe), (b) purified hydrogel, (c) separated ACNF gel, (d) unidirectional aerogel after ice-templating, and (e) after CaCl_2_ crosslinking. (f) Photograph of ACNF-X aerogel and illustration of aerogel sample detailing the longitudinal and transverse directions.

The preparation process of the seaweed-inspired aerogels studied in this work is summarized in Figure 1. The seaweed stipe (Figure 1a) was purified to remove pigments and impurities, while preserving the alginate and cellulose content in the resulting natural hydrogel structure (Figure 1b). After purification, the fibrillation process resulted in the separation of the cellulosic part into very fine nanofibers through the shearing forces applied to the sample by ultrafine grinding, resulting in a gel composed of alginate and CNFs (Figure 1c). The characterization of the gel structure was reported in our previous study,^23^ and the gel composition is summarized in the Materials section. The ACNF gel was then ice-templated and freeze-dried to form an anisotropic ACNF aerogel structure (Figure 1d) and treated with solutions of CaCl_2_ in ethanol to generate a crosslinked sample (ACNF-X) (Figure 1e,f).

### Aerogel Structural Stability

**Figure 2:**
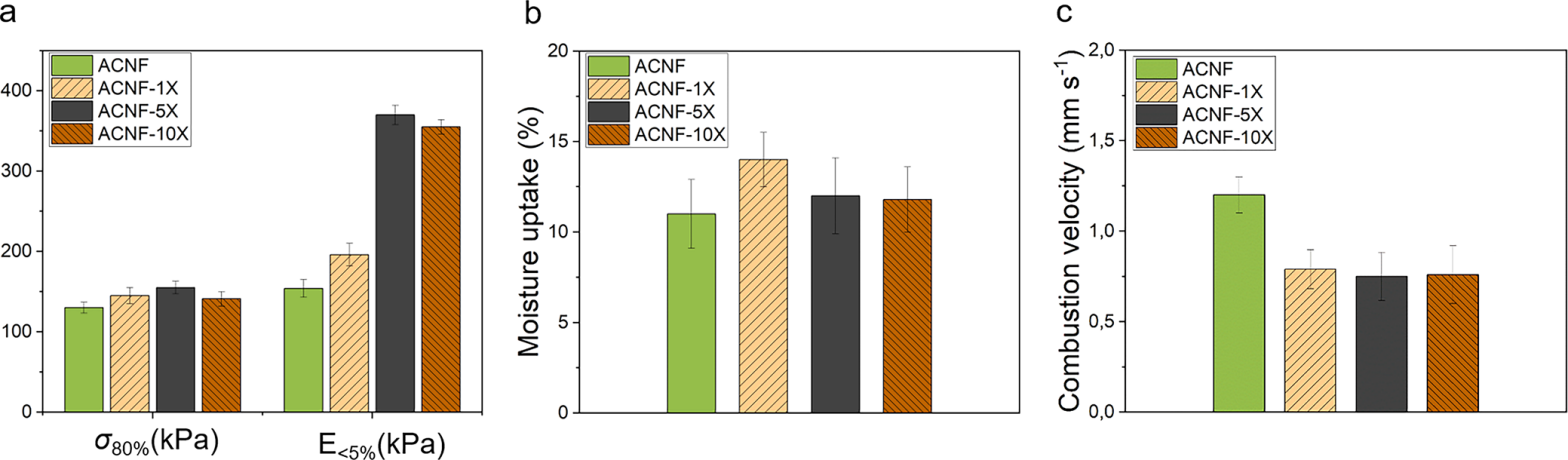
(a) Compressive strength at 80% strain (σ_80%_) and compressive modulus (*E*_<5%_) after compression testing in the longitudinal direction. (b) Moisture uptake at 75% relative humidity. (c) Combustion velocity determined from videos recorded during the horizontal flame test.

Ethanol–CaCl_2_ solutions were prepared in various concentrations (1, 5, and 10 wt %) to establish the most favorable crosslinking conditions by initial screening of the aerogel’s structural stability. The structural stabilities arising from different crosslinker concentrations were compared according to their mechanical properties, moisture uptake, and flame-retardant behavior, which are key characteristics for their use in insulation applications, as presented in Figure 2.

From Figure 2a, the highest compressive modulus (370.3 ± 12.8 kPa) was achieved at a crosslinker concentration of 5 wt %, which corresponds to an increase of more than 140% compared to that of the non-crosslinked sample (154.2 ± 11.2 kPa). Meanwhile, the strength of the aerogels appeared to be only slightly affected by the crosslinking and its concentration; the samples demonstrate comparable behavior, as shown in Figure 2a and the representative stress and strain curves in Figure S2. Because the initial mechanical testing was carried out along the freezing direction (longitudinal direction) of the aerogels, the aligned nanofibers contributed to the mechanical stability of the aerogel structure. The crosslinking of this structure had the largest influence on the initial compression of the aerogel, while after the plateau and densification stages, the behavior was comparable. As a hydrophilic polymer rich in hydroxyl and carboxyl groups, alginate (and cellulose) aerogels easily absorb moisture even after careful drying.^22^ The CaCl_2_ crosslinking of alginate has been shown to affect the absorption of water.^25^ Moisture uptake, or rather, moisture resilience, is an important characteristic of aerogels for insulation applications; moisture adsorption was also evaluated for various CaCl_2_ concentrations as a screening parameter of the aerogel’s structural robustness. From the moisture absorption data, it was found that the concentration of CaCl_2_ had a minor effect on moisture uptake (Figure 2b) and that the crosslinking process did not lead to an increase in the overall moisture uptake of the aerogels.

The flame-retarding performances of ACNF and ACNF-X at different CaCl_2_ concentrations were determined by a horizontal combustion test (Figure 2c). The average combustion velocity of ACNF was 1.2 ± 0.1, while a lower burning rate was observed after crosslinking for all aerogels—as low as 0.75 ± 0.1 mm s^–1^ (ACNF-5X). The flameless combustion behavior slowly consumed the ACNF aerogel, leaving behind an uncollapsed, shrunken char residue. In contrast, all crosslinked aerogels were only partially consumed and displayed a self-extinguishing behavior. Fire-retardant behavior was observed at 1 wt % crosslinking, and increasing the concentration did not further suppress the flammability. This behavior is in agreement with previously studied alginate aerogels, which were found to exhibit non-flammability and only smoldering behavior during combustion.^22^ In contrast, CNF aerogels produced from wood pulp have been reported to ignite extremely quickly with an average combustion velocity of 58.4 mm s^–1^^18^ This demonstrates the tremendous potential of CNF isolated from alginate-rich seaweed as a material with natural flame retardancy, which can be further enhanced with crosslinking.

The aerogels crosslinked with 5 wt % CaCl_2_ solution exhibited the most favorable mechanical properties, while remaining unaffected in terms of moisture uptake and flame-retardant behavior. Thus, the ACNF-5X aerogel was selected, along with the ACNF aerogel as a reference sample, for further study according to the unidirectional ice-templating and crosslinking effect on the structural behavior and thermal performance.

### Aerogel Structure

**Figure 3:**
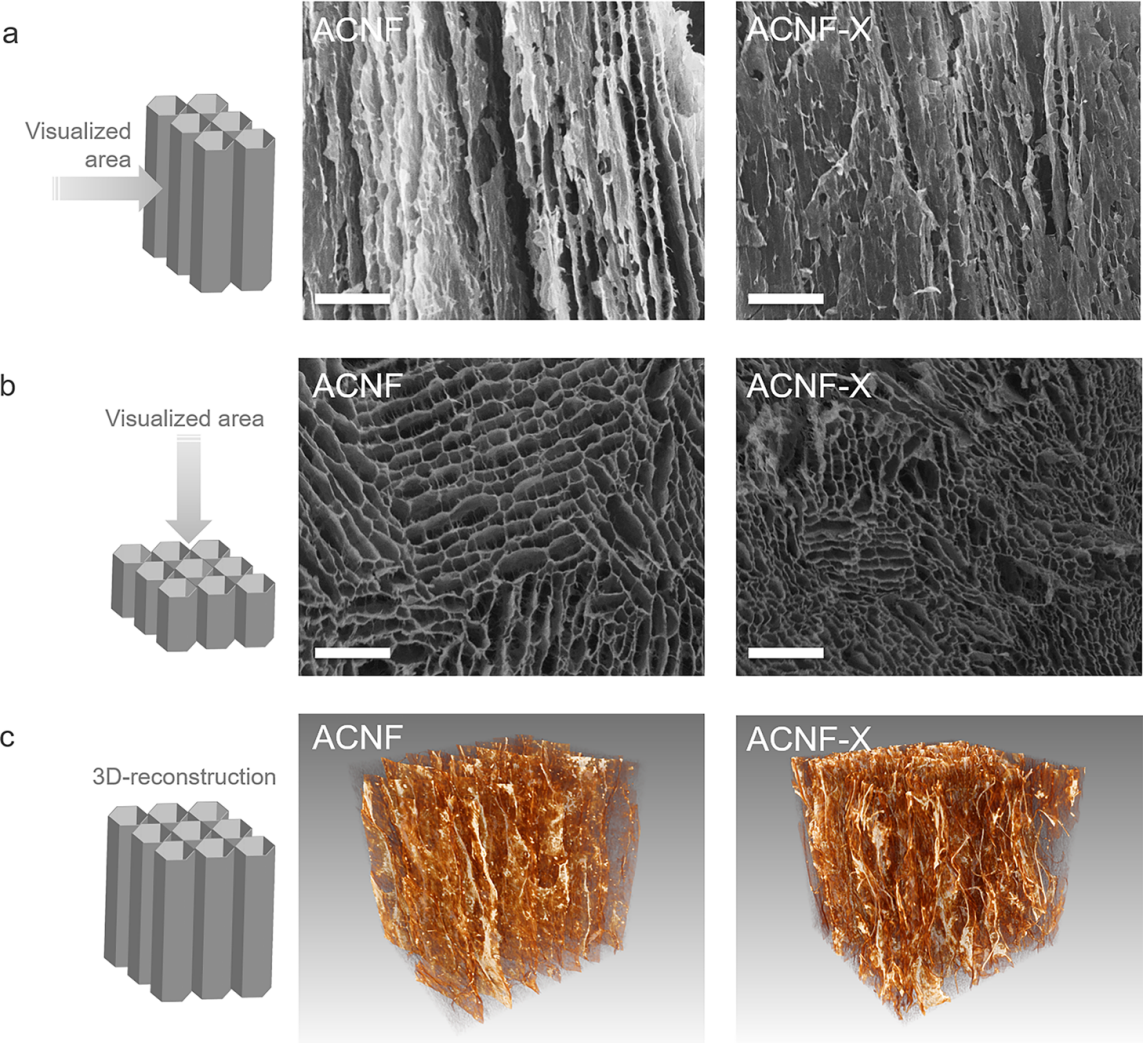
Morphological appearance of ACNF and ACNF-X aerogels. SEM micrographs along the freezing (longitudinal) direction (a) and cross section (b). Scale bar: 500 μm. XRT 3D reconstruction (c).

The morphology of the ACNF aerogels after ice-templating and crosslinking was studied by scanning electron microscopy (SEM) and 3D reconstruction using X-ray tomography (XRT); the results are shown in Figure 3.

The aerogels exhibited an anisotropic porous structure generated by the ice-templating process with an obvious orientation that followed the direction of ice-crystal growth, that is, along the freezing (longitudinal) direction (Figure 3a). The elongated channels generated in the ice-crystal growth direction can provide rapid energy transfer in the longitudinal direction, which can be beneficial for applications such as thermal insulation.^14^

As shown in Figure 3a,b, following ionic crosslinking by Ca^2+^, the aerogel structure formed after the ice-templating and sublimation was still preserved, yet it was composed of compact pores with a narrower pore diameter ranging from 40 to 280 μm compared to 60–400 μm for the ACNF aerogel (Figure 3a,b). A higher flame velocity has been reported for porous materials with larger pores,^26^ which is in agreement with the suppressed flame propagation upon crosslinking. The structure resembled that of wood-based CNF aerogels assembled using the same approach at 1 wt % solid content,^27^ indicating that the presence of alginate does not hinder ice-crystal growth. This is confirmed by the images of the 3D reconstructions obtained by XRT scanning, as shown in Figure 3c. The aerogels show distinct orientations along the freezing direction, yet the channel-like structure of the aerogel appeared narrower and less structured after crosslinking, which likely influenced the overall pore tortuosity.

**Figure 4:**
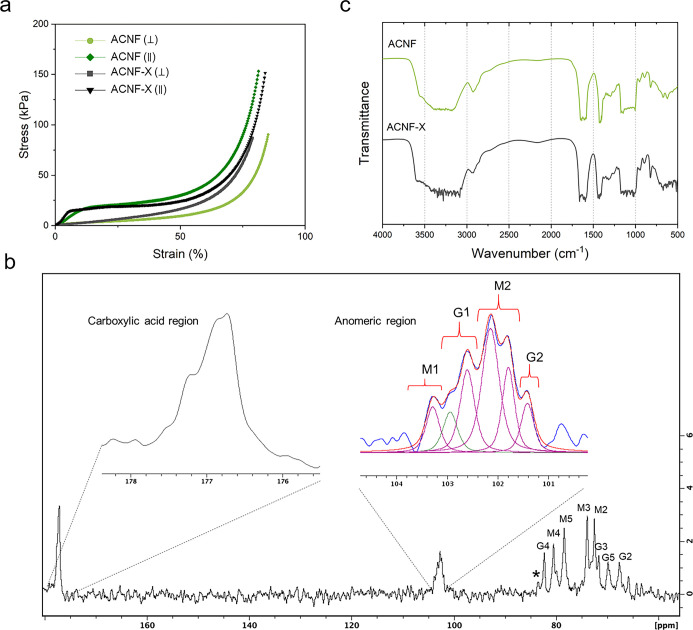
(a) Representative stress–strain curves of ACNF before and after crosslinking tested in both directions and (b) ^13^C NMR spectrum and signal assignment of alginate (ACNF sample), where impurities are marked with *. (c) FTIR spectra of ACNF and ACNF-X.

The performance and anisotropy of the aerogels after crosslinking were further studied and compared with those of the non-crosslinked samples. Table 1 summarizes the effect of ice-templating and crosslinking on the physical properties of the aerogels, and Figure 4 presents the representative stress–strain curves and chemical structure based on the analysis of the NMR and FTIR spectra. The non-crosslinked aerogels displayed very low densities and relatively low surface areas, as shown in Table 1. The samples gain density but still persist at very low levels, while the surface area displayed comparable values upon crosslinking.

**Table 1:** Physical Properties of the Aerogels and Compressive Modulus (*E*_<5%_) and Stress σ (at 20 and 40% Strain) Conducted in Both the Longitudinal (∥) and Transverse (⊥) Freezing Directions before and after CaCl_2_ Crosslinking at 5 wt %

sample code	density (kg dm^–3^)	BET surface area (m^2^ g^–1^)	*E*_<5%_ (kPa)	σ_20%_ (kPa)	σ_40%_ (kPa)
ACNF (⊥)	0.012 ± 0.001	19.5 ± 3.2	25.2 ± 4.1	5.1 ± 0.7	8.3 ± 0.9
ACNF (∥)			154.2 ± 11.2	21.1 ± 1.0	24.3 ± 1.3
ACNF-X (⊥)	0.015 ± 0.001	25.9 ± 4.1	41.5 ± 5.1	6.3 ± 0.5	12.5 ± 1.1
ACNF-X (∥)			370.3 ± 12.8	20.8 ± 1.3	23.9 ± 1.4

In general, as the crosslinking density of the polymer increases, the surface area also increases,^28^ which can affect the thermal conductivity.^29^ The surface area of the seaweed-derived ACNF aerogel can be compared to that of wood-derived CNF aerogel using the same assembly approach and dry content, namely, 19.5 m^2^ g^–1^ compared to 10.4 m^2^ g^–1^. At these ranges, no difference in the surface area can be determined. In addition, the compressive strengths at 20 and 40% strain were comparable to those of wood-derived CNF aerogels.^27^

The increase in compressive properties after crosslinking was more pronounced than the increase in densities. The aerogel stiffness increased by as much as 800% with unidirectional ice-templating, while the crosslinking simultaneously contributed to the stiffness in both the longitudinal and transverse directions (Table 1).

As shown in Figure 4a, the compressive stresses of the aerogels increased slowly up to approximately 60% strain, followed by a steep increase in stress. This behavior is consistent with a classic foam-like material, in which the cellular structure of an aerogel collapses when the applied stress induces a large strain, resulting in a closely contacted network skeleton.

The linear chains of alginate are composed of different blocks of guluronic and mannuronic acids (i.e., MM, GG, MG, or GM blocks), and the linkage in the block structure determines not only the flexibility in the alginates but also its crosslinking ability.^30^ The GG blocks are responsible for forming ionic complexes in the presence of Ca^2+^, which generate a stacked (crosslinked) structure known as the “egg-box model”; therefore, alginates with lower M/G ratios display a higher affinity toward ionic crosslinking.^31^ To evaluate the chemical structure of the alginate in the ACNF aerogel and its influence on the crosslinking behavior, a ^13^C NMR spectrum was acquired (Figure 4b), and the signals were assigned in accordance with the literature.^32−36^ FTIR spectra were then obtained for both ACNF and ACNF-X (Figure 4c) to study the crosslinking in detail.

The ratio between mannuronate (M) and guluronate (G) units was determined by line-shape fitting-based integration of signals in the ^13^C NMR spectra (Figure 4b). The signals of the anomeric region (C1) were grouped according to previous studies as follows: M1: MMG and GMG, G1: MGG and GGG, M2: GMM and MMM, and G2: MGM and GGM.^32,33^ The wide signals from the ring carbons C2–C4 (65–85 ppm) of the M and G units indicate that the chains are heteropolymeric (Figure 4b). There are also some impurities from proteins and cellulose residues, which are marked with an asterisk (*) in Figure 4b. Anomeric C1 carbon (101–104 ppm) is the most affected by variation of the M–G block and provides information about the polymer linkage.^32,34^ The carboxylic acid group C6 (176–178 ppm) is less affected and does not provide detailed information on the linkage.^33^ The M/G ratio was determined by integrating the ring carbons (C2–C4) and anomeric (C1) regions in the spectrum, resulting in M/G = 1.6. The integration of the signals from the ring structures (C2–C4) resulted in a slightly higher value of M/G = 2.0. The M/G ratio of 1.6–2.0 is in agreement with that reported for commercial alginate.^21^

The FTIR spectra of the ACNF and ACNF-X samples are shown in Figure 4c. The broad peak centered at ≈3250 cm^–1^ and the smaller peak at 2926 cm^–1^ can be attributed to O–H and C–H stretching, respectively, and are typical of polysaccharides,^37−40^ supporting the presence of both alginate and CNFs in the aerogels. The C–H peak shifted slightly to 2922 cm^–1^ and was less intense in the crosslinked sample. The peak appearing at around 1650 cm^–1^ arises from the presence of water.^38,41^ The absorption band at 1607 cm^–1^ can be assigned to asymmetric COO^–^ stretching vibrations^37,38,42^ and is found at 1595 cm^–1^ in the ACNF-X spectrum, a shift associated with an increase in the strength of the ionic interaction owing to Ca^2+^ crosslinking.^38^ The band at 1420 cm^–1^ can be attributed to the C–OH deformation vibration and COO^–^ symmetric stretching,^37−39,42^ and the shift of this band to 1440 cm^–1^ in the ACNF-X spectrum can also be attributed to ionic crosslinking.^40^ The peak centered at ≈1300 cm^–1^ is related to C–C–H and O–C–H deformation^39^ and C–O stretching of the polysaccharide chains,^38^ while those at approximately 1000–1200 cm^–1^ are related to C–C, C–O, and C–O–C stretching in the polymer backbones.^37,41^ The bands at ≈950 and ≈890 cm^–1^ can be assigned to C–O stretching and the C1–H deformation vibration of β-mannuronic acid residues, respectively.^39^ The band appearing at 817.8 cm^–1^ is also related to mannuronic acid.^43^

### Thermal Performance for Insulation Applications

**Figure 5:**
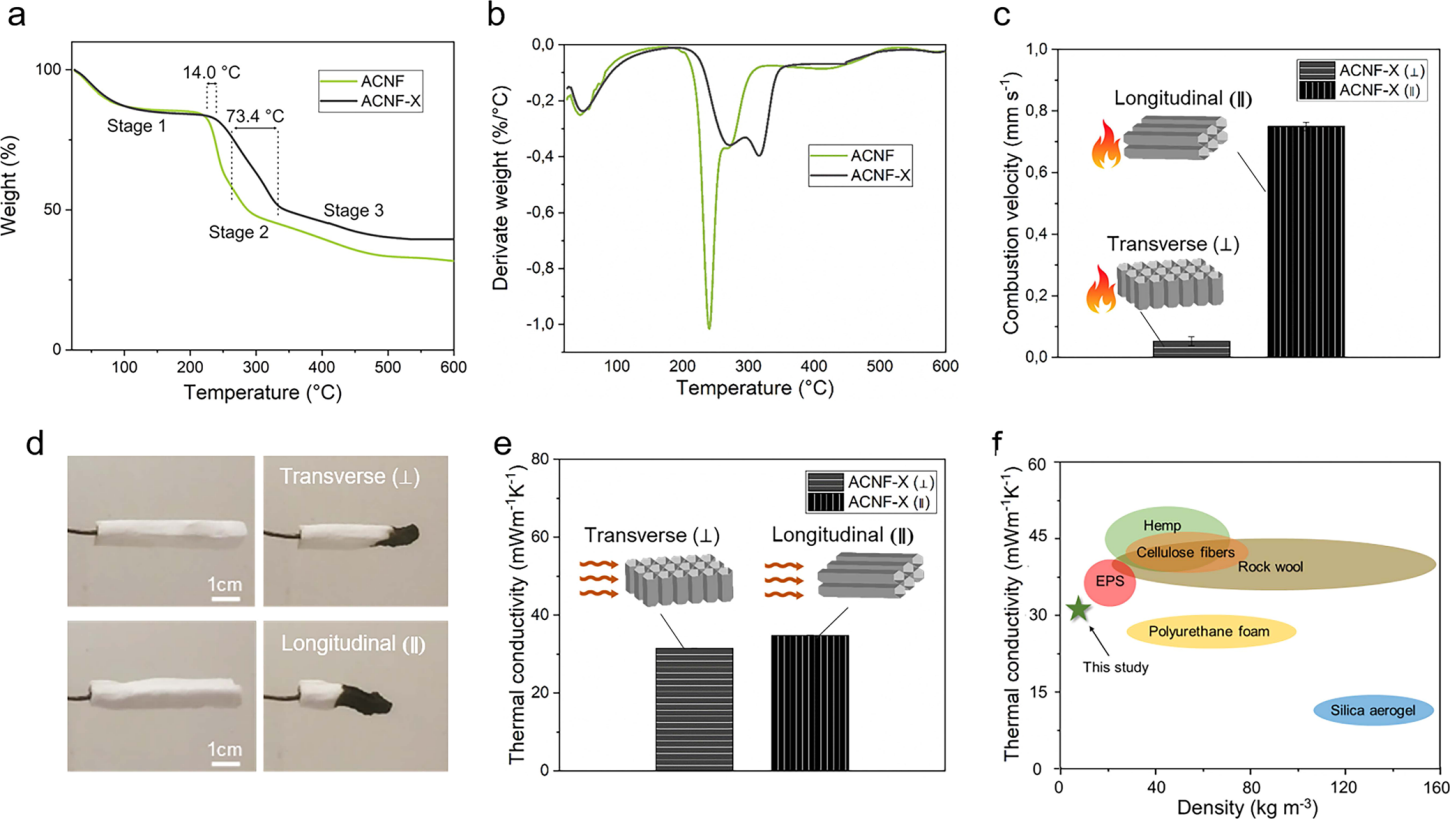
(a) TGA and (b) derivative thermogravimetric curves of ACNF and ACNF-X aerogels. (c) Combustion velocities from the horizontal flame test showing where the flame was applied on the sample and the resulting longitudinal and transverse directions of flame propagation. (d) Photographs of aerogels before and after 60 s combustion in the different sample directions. (e) Thermal conductivity (λ) values of ACNF-X aerogels measured in the transverse and longitudinal directions. (f) Ashby plot of thermal conductivity as a function of density according to Pfundstein et al.^3^ and Aravind et al.^44^ for commonly used insulation materials, Koebel et al.^45^ for silica aerogels, and the results if this study.

Thermal stability, insulation performance, and combustion behavior are important characteristics of aerogels for insulation applications. Figure 5 summarizes the thermal properties according to the degradation behavior, combustion velocity, and thermal conductivity of the aerogels, including the effect of crosslinking and the aligned structure.

The first stage of the degradation process is related to the loss of water and is common to hydrophilic and hygroscopic materials such as alginate and cellulose.^21,22^ The weight loss during this stage was 15.0 and 16.2% for the ACNF and ACNF-X samples, respectively.

The second stage can be attributed to the degradation of the polymer chains of both cellulose and alginate.^21^ The onset temperature of this degradation stage is higher for the crosslinked sample, at 227.4 °C for ACNF and 241.4 °C for ACNF-X. This indicates that the crosslinked structure is more thermally stable than the non-crosslinked structure. An onset temperature of 222.0 °C has been reported for neat Ca^2+^-crosslinked alginate aerogels.^46^ In the same study, the addition of TEMPO-oxidized CNFs to the alginate aerogels was found to increase the degradation temperature by 15.4 °C, resulting in a value similar to that observed in the current study for the crosslinked ACNF aerogels. However, Lin et al.^46^ reported no effect when using cellulose nanocrystals or unmodified CNFs. Weight loss during the second stage was similar for both samples; 34.6 and 33.6% losses were observed for ACNF and ACNF-X, respectively. However, the end-point temperature of the second stage is significantly higher for the crosslinked sample (334.8 °C) in comparison to the uncrosslinked sample (261.4 °C). The third stage is related to the degradation of carbon-based residues^21^ and also has a higher end point in the case of crosslinked sample. The residual weights of the ACNF and ACNF-X samples were 31.7 and 34.6%, respectively.

From Figure 5c, the combustion velocity in the longitudinal direction is very low at 0.75 mm s^–1^ and is reduced by about 15-fold in the transverse direction to 0.053 mm s^–1^, signifying that ice-templating of the aerogel structure could be beneficial in reducing the flame retardancy of porous materials. From Figure 5d, it can be seen that not only was the combustion velocity slower in the transverse direction but the flame propagation and the aerogel self-extinguished sooner in the transverse direction compared to the longitudinal direction. Combustion is generally initiated in the presence of flames with oxygen taken from the surroundings. Prior to combustion, the materials undergo thermal degradation, where some of which is degraded turns into combustible, volatile products and combined with oxygen, which can fuel the flame.^47^

Although the anisotropic structure was preserved after crosslinking, the distinctly aligned channels appeared less structured after crosslinking, as shown in Figure 3. Despite this, the architecture in the transverse direction appears to favor the degradation pathways, which lead to the production of carbonaceous residues rather than combustible volatile gases via either re-radiating the heat or slowing down the heat transmission and volatile diffusion.^47^

Apart from the aerogel structure, the predominately flame-retardant behavior of ACNF-X may be attributed to two reasons. First, generation of vapor and CO_2_ by dehydration and decarboxylation carry away most of the heat during alginate decomposition process, where alginate molecules breaks down into M and G blocks.^48^ Meanwhile, in the outermost layer, the remaining solid residue forms a dense carbon layer. The formation of the barrier layer can prevent heat and combustible gas from diffusing into the inner layer, notably reducing the heat release rate and promoting the flame retardancy.^22,47^ Second, Ca^2+^ may promote the formation of a denser barrier char layer that suppresses the flame propagation. Formation of a thick residue crust containing carbonaceous char and calcium carbonate has been shown after combustion of Ca^2+^ crosslinked alginate fibers.^17^ In the same study, it was reported that calcium alginate produces H_2_O and inert CO_2_ through decarboxylation, which decreases the combustible gas concentration and hinder the heat transfer, thus contributing to the flame-retardant mechanism.

The thermal conductivity (λ) measurements were compared at *t* = 0 h for the ACNF-X samples. However, it was noted that longitudinal samples showed stable results after 2 weeks of measurement, which could be attributed to their low compressibility. On the other hand, the transverse samples showed compressibility, which induced an artifact over time; therefore, the samples were compared at *t* = 0 h (Table S1).

From Figure 5e, aerogels aligned in the longitudinal direction exhibit λ values of 34.8 ± 0.2 mW m^–1^ K^–1^, while those aligned in the transverse direction have values of 31.5 ± 0.4 mW m^–1^ K^–1^. This difference is outside the error and standard deviation and indicates that the alignment of the fibers has an effect on λ. Although this difference is significant and reproducible (*t*-test: *p* = 0.95, *n* = 4), it is small in comparison to the halving of λ seen in other studies.^11,12^ However, a trend exists in which the transverse samples show approximately 10% lower λ than their corresponding longitudinal counterparts. For alginate aerogels, our measured λ lies in a similar range as those reported in other studies (26–50 mW m^–1^ K^–1^).^22,49^ For biopolymer-based aerogels, pectin and starch carbohydrate aerogels show thermal conductivities in the range of 18–22 mW m^–1^ K^–1^,^50,51^ and cellulose-nanofiber-based aerogels exhibit values of 18–30 mW m^–1^ K^–1^ for varying densities.^9^ However, these aerogels were all dried using supercritical CO_2_, resulting in a completely different microstructure, and incorporated additional processing steps that add to the environmental impact.

A reduction in the thermal conductivity could be realized by reducing the mean-free path of air. This could be realized by an optimized microstructure and reduced path of heat flow, as well as altering the predominantly macroporous network. The mechanical compression of samples has been shown to be an easy and scalable process for reducing thermal conductivity. Along with its low cost, λ is shown to have a significant difference in both the transverse and longitudinal directions.^9,52^ To determine how this approach could affect the ACNF-X samples, the samples were uniaxially compressed to 50% of their initial height, which resulted in a λ value of 28.2 ± 0.1 in the transverse direction (Table S1).

As shown in Figure 5f, the ACNF-X aerogels exhibit a λ value competitive with traditional thermal insulation materials with values of 30–50 mW m^–1^ K^–1^, such as EPS, PU foam, and rock wool, as well as natural insulation materials such as cellulose fibers and hemp.^3^ Silica aerogels have a highly mesoporous isotropic network that contributes to the performance of the industry-leading aerogel (λ = 12–15 mW m^–1^ K^–1^).^44,45^ However, their manufacturing process includes the use of hazardous solvents, supercritical drying, and high pressure and temperature, combined with an expensive precursor that limits their applications in many fields.^8^

Among these thermal insulation materials, hemp is an organic, natural insulating material that is untreated and can easily be recycled, but its flame-retardant, polyester fibers complicate the composting and recycling options.^3^ Synthetic insulation materials, such as EPS, are largely employed in construction and occupy a considerable market share today, owing to their insulation, low cost, and non-hygroscopic properties. However, because of its high flammability, it also includes flame-retarding agents that produce harmful substances during combustion, causing concerns to human health and the environment. When EPS insulation is removed from buildings, the recycling options are very limited because of the residues of mortar and other building chemicals.^3^ The thermal conductivities of the seaweed-based and flame-retardant aerogels prepared in this work are comparable to those of EPS and PU foams (Figure 5f) and are highly competitive from the perspective of sustainable building industry.

### Outlook and Limitations of Seaweed-Derived Aerogels

This study focused on the use of completely bio-based resources, targeting efficient upscalable conversion processes with low environmental impact. Separation of nanofibers rich in alginate directly from seaweed, combined with ice-templating and freeze-drying, followed by Ca^2+^ crosslinking can offer an eco-efficient approach from the raw material to the high-performance aerogel. This, in comparison to the multiple processing steps, including chemicals and energy, is needed for the extraction of alginate and CNFs separately,^10,31^ or to existing aerogel assembly methods.^53^ However, a life cycle assessment would be required to establish the environmental impact from seaweed to aerogel. From an environmental perspective, the largest contributors to the environmental burden of nanofiber production have been identified as chemicals and energy.^54^ Nanofibers separated from seaweed have specific functional properties owing to the presence of alginate, at the same time; their yield is very high at 71 wt %. This is achieved by applying direct bleaching prior to nanofiber separation instead of the multiple purification steps commonly used for cellulose purification, including washing and alkali treatment. Furthermore, the measured energy consumption for their mechanical separation process is remarkably low (1.0 kW h kg^–1^); meanwhile, the commercially bleached wood kraft pulp consume 8.4 kW h kg^–1^ for a similar mechanical processing.^23^ In a previous study, we have systematically evaluated the approach of direct bleaching, followed by mechanical separation of industrial residue.^54^ This resulted in a reduced environmental impact of more than 75% for carbon footprint, along with a cost reduction of more than 50%, owing to an increased yield and reduced energy, chemical, and water use.

In order to put the use of seaweed-based CNFs in a broader context for thermal insulation applications, a different protocol for drying was also investigated to provide an idea of how that could affect the thermal conductivity because supercritical drying is known to preserve the pore network and structure better than freeze-drying. Therefore, the ACNF aerogels were also dried with supercritical CO_2_. They showed 15 and 22% lower thermal conductivities than the transverse and longitudinal samples, respectively, yet it should be noted that their densities were higher and the specific surface area was measured to be about 200 m^2^ g^–1^. The application potential of seaweed-derived aerogels can thus be expanded by applying different processing techniques to achieve different industrial requirements. Upon replacement of industrial insulation systems, it would be highly advantageous, from a sustainability perspective, if the materials used to replace the existing insulation systems are biodegradable, and the source of the raw materials are renewable and bio-based. Another important emphasis for hygroscopic, bio-based aerogels is the influence of moisture on their properties, such as thermal conductivity.^16^ Thus, there is a need to investigate the relative humidity dependence of the thermal conductivity of seaweed-derived aerogels in detail. Bio-based flame retardants represent a promising direction for designing next-generation insulation materials, and another key aspect in this development is to utilize the intrinsic properties that seaweed offers, as highlighted in this study.

## Conclusions

Flame-retardant and heat-insulating aerogels were successfully developed from seaweed by isolating CNFs rich in alginate, which act as a natural flame-retarding agent. This resource- and eco-efficient approach of isolating nanofibers was combined with ice-templating and non-toxic CaCl_2_ crosslinking to produce a natural anisotropic aerogel with multifunctional properties such as lightweight, good mechanical properties, high thermal stability, low thermal conductivity, considerable flame retardancy, and self-extinguishing behavior. The assembly of an anisotropic aerogel structure and its crosslinking considerably improved the intrinsic characteristics by reducing the combustion velocity by almost 96% of ACNF-X compared to ACNF measured in the longitudinal direction. Simultaneously, the mechanical properties of the crosslinked samples displayed 800% enhanced stiffness upon alignment. Meanwhile, the anisotropic structure also influenced the flammability and thermal conductivity, which were reduced by 93 and 10%, respectively, in the transverse direction. It was shown that the thermal conductivity could be further reduced by 11% by mechanically compressing the aerogels, resulting in a λ value of 28.2 mW m^–1^ K^–1^. This three-step approach for the development of a multifunctional green insulation material incorporates renewable resource efficiency and facile and environmentally benign aerogel assembly without the need for expensive or harmful additives. Aside from the environmental perspective, this study highlights the benefits of preparing aerogels from seaweed-based CNFs for insulation applications. These aerogels possess inherent characteristics that could be utilized as a template for the future development of next-generation green insulation materials.

## Supporting Information Available

Photographs of aerogel samples; representative stress and stain curves of ACNF, ACNF-1X, ACNF-5X, and ACNF-10X, tested in the longitudinal direction; N_2_ adsorption of ACNF and ACNF-5X; enlarged representative stress and stain curves of ACNF and ACNF-X tested in longitudinal and the transverse directions; and summarized λ values measured at different times (PDF)
