# Supplementary material for: Seaweed-Derived Alginate–Cellulose Nanofiber Aerogel for Insulation Applications
Source: ACS Appl Mater Interfaces. 2021 Jul 13;13(29):34899–909. doi: 10.1021/acsami.1c07954 (PMC8323098; doi:10.1021/acsami.1c07954)
Supplement: Supplementary file 1 — am1c07954_si_001.pdf [file am1c07954_si_001.pdf]

Supporting Information

Seaweed-Derived Alginate-Cellulose Nanofiber Aerogel for Insulation Applications

Linn Berglund[*a], *Tuukka Nissilä*[b], *Deeptanshu Sivaraman*[c], *Sanna Komulainen*[d], *Ville-Veikko Telkki*[d], *Kristiina Oksman* [a,e]

[a] Division of Materials Science, Luleå University of Technology, 97187, Luleå, Sweden

[b] Fibre and Particle Engineering, University of Oulu, Oulu, Finland

[c] Empa – Building energy materials and components, Swiss Federal Laboratories for Materials Science and Technology, Dübendorf, Switzerland

[d] NMR Research Unit, University of Oulu, Oulu, Finland

[e] Mechanical & Industrial Engineering, University of Toronto, Canada

*Corresponding author

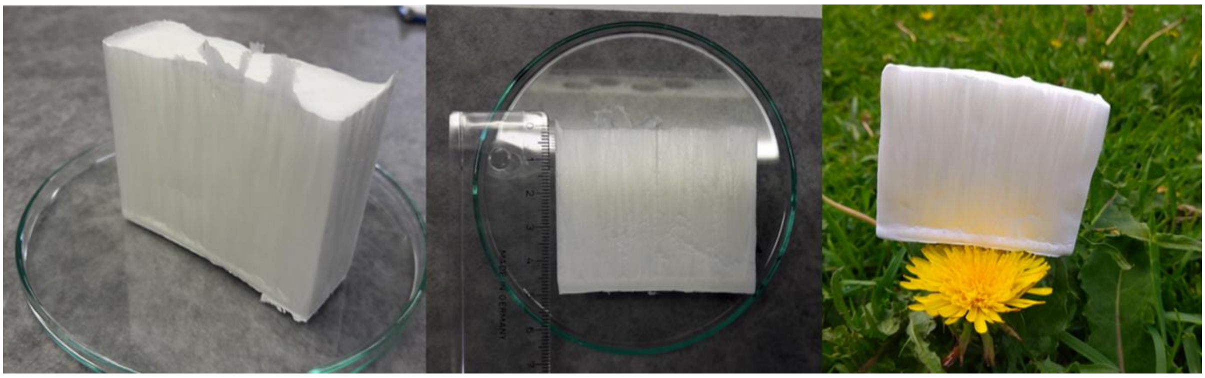

Figure S1. Photograps of the aerogel.

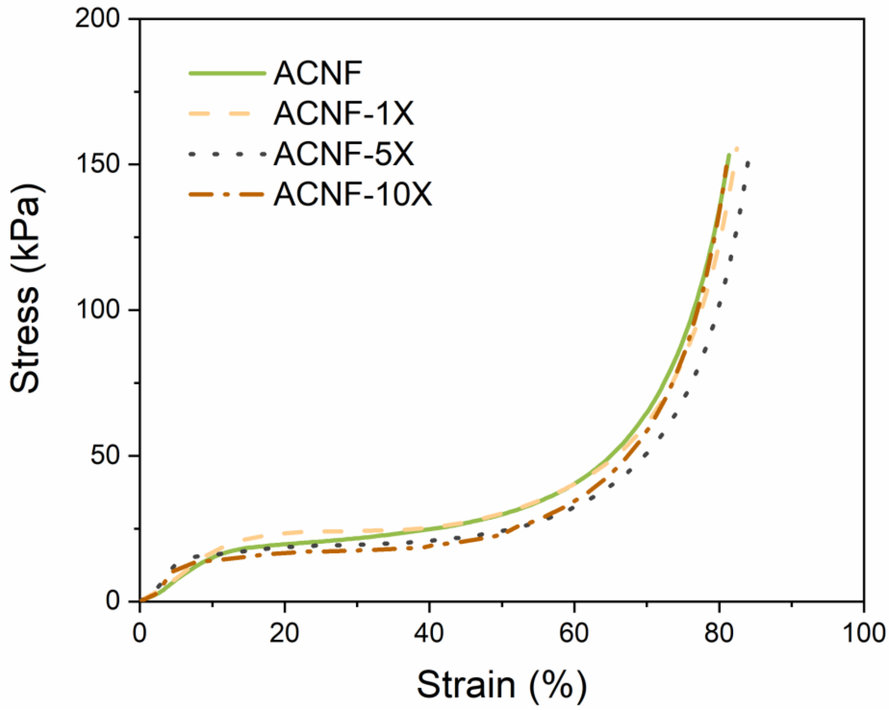

Figure S2. Representative stress and stain curves at different $CaCl_2$ crosslinking concentrations.

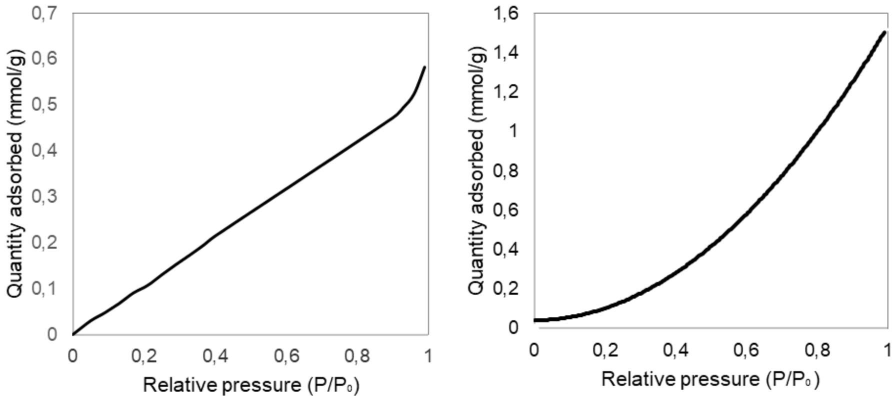

Figure S3. N_2 adsorption of ACNF (to the left) and ACNF-X (to the right).

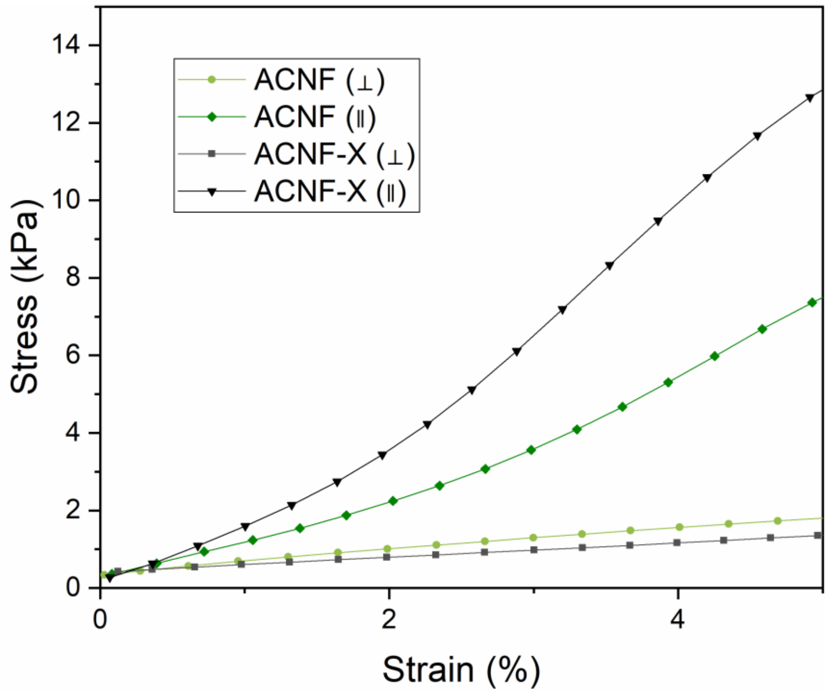

Figure S4. Enlargement of the representative stress and stain curves of ACNF before and after crosslinking, tested in both directions (Figure 4a).

Table S1 Summarized values of thermal conductivity mesurments at different times.

**Sample**	**0h**	**2 Weeks**	
**ACNF (//)**	34.8 ± 0.2	34.6 ± 0.2	(n=4)
**ACNF-X (⊥)**	31.5 ± 0.4	31.7 ± 0.5	(n=4)
**ACNF-X(⊥) Compressed**	28.2 ± 0.1	29.0 ± 0.5	(n=2)
**ACNF Supercritical dried**	26.9 ± 0.50		(n=2)
